# Integrating pathology into human disease modelling – how to eat the elephant

**DOI:** 10.1242/dmm.016394

**Published:** 2014-05

**Authors:** Cheryl L. Scudamore

**Affiliations:** Mary Lyon Centre, MRC Harwell, Oxfordshire, OX11 0RD, UK.

**Keywords:** Pathology, Phenotyping, Mouse, IMPC

## Abstract

Mouse models are increasingly being used for the study of human disease, and the generation and functional characterisation of new models is underpinned by high-throughput phenotyping consortia such as the International Mouse Phenotyping Consortium. A new study by Adissu and colleagues, published in *Disease Models & Mechanisms*, demonstrates the usefulness of histopathology in providing corroborative information and uncovering novel phenotypes in genetically modified mice in a high-throughput screen. Although pathology is recognised as a valuable tool to enhance our understanding of animal disease models, it has also been systematically under-resourced. This Editorial aims to highlight ways in which the gap between the usefulness of pathology and its perceived inaccessibility can be addressed by considering pragmatic solutions for planning, resourcing and accessing pathology expertise. The role of funding agencies, academic centres and journals in ensuring that the value of pathology is fully recognised and is adequately supported and funded is also discussed.

“When eating an elephant take one bite at a time” –

Creighton Williams Abrams.

## Pathology: an underexploited resource

The usefulness of pathology in the study of human disease using mouse models has been repeatedly extolled in recent years; however, there have been parallel warnings of an apparent lack of available resources in this area (Warren et al., 2009). A new study by Hibret Adissu and colleagues from the University of Toronto in collaboration with researchers involved in the Sanger Mouse Genetics Project highlights the value of pathology in confirming the results of *in vivo* phenotyping tests and identifying additional phenotypes not revealed in high-throughput mouse phenotyping pipelines such as the International Mouse Phenotyping Consortium (IMPC) (Adissu et al., 2014). In the study, which is published in this issue of *Disease Models & Mechanisms*, histopathology was added to the assessment of 50 randomly chosen mouse lines from the IMPC programme. Their results show that histopathology was beneficial in providing confirmatory morphological data for 63% (19 out of 30) of lines in which the standard primary screens had detected a phenotype, but, more importantly, it also detected novel phenotypes (not detected or predicted from the initial screen) in 14% of the lines (7 out of 50). The authors also highlight the value of using an expert pathologist who can rule out ‘incidental pathology’ (i.e. related to the background strain or environment and not the genetic modification) by a combination of comparison to control animals and prior experience. Thus, the study demonstrates that the incorporation of pathology into a high-throughput mouse phenotyping screen can validate data provided in the primary screen and also yield valuable new information.

Pathology examination of tissues has been recognised as a particularly valuable primary end point in safety studies in the pharmaceutical industry for many years, and no new medicines would pass into human clinical trials without this end point being explored (ICH, 2009). In this crucial step, a range of biological parameters are integrated with the morphological observations obtained, and the value of pathologists in both acquiring the pathology data and interpreting it is well recognised. Good pathology practices also make a substantial impact to the 3Rs (**R**educing the number of animals used, **R**efining experiments to minimise the impact on animals, and **R**eplacing animal experiments wherever possible with alternatives) by maximising the data gained from individual animals and therefore reducing the number of animals needed to fully evaluate a model or phenotype. Despite the evidence for the utility of laboratory animal pathology in the drug discovery process (Jubb et al., 2014), for a number of reasons it is still common for pathology not to be routinely included in animal studies outside the pharmaceutical industry. Some of these reasons are discussed by Adissu et al., including the cost of the process, lack of standardised protocols and the lack of available comparative pathologists (Adissu et al., 2014). This can lead to three common missed opportunities: no pathology is attempted at all; pathology is attempted but, because of lack of planning, no results can be generated; or inexperienced researchers attempt their own pathology leading to interpretive errors (some of which find their way into the scientific literature). The lack of expert mouse pathologists in academic environments does not seem to have significantly improved in the last 10 years (Sundberg et al., 2004; Adissu et al., 2014), but the number and complexity of mouse models being created continues to expand at an ever-increasing rate. It is thus of crucial importance for the intrinsic value of pathology to be recognised and adopted in routine laboratory practice. But how do we eat the (pathology) elephant and ensure that this rich dataset is included in future studies (Brown and Moore, 2012)?

## Planning for pathology end points

To the non-specialist, pathology can seem like a daunting and unachievable end point to include in a study. The basic techniques involved in cellular pathology evolved over 150 years ago (reviewed in van den Tweel and Taylor, 2010) and, although there have been technical advances, particularly in optics, many of the methods remain strikingly similar and require relatively simple equipment. Although the techniques might appear unfashionably ‘low-tech’, they have the advantage of being robust, well understood and validated by long-term use. In short, anatomic pathology has stood the test of time as a useful tool in helping to build our understanding of disease by linking morphology to pathogenesis. Even in the genomic era, the whole animal overview provided by systematic pathology is vital to gaining a holistic view of phenotypes in young (Adissu et al., 2014) and ageing (Ladiges et al., 2013) mice.

The first stage to incorporating pathology as an end point is to plan to do it, i.e. build it into grant applications and subsequent experimental protocols. As mentioned, the cost of pathology is often held up as an argument against its use, but it is important that cost-benefit analysis is properly performed. Adissu and colleagues demonstrate that a primary screen for pathology phenotypes can be effectively performed using a small cohort of mice (two males and two females) and, in an on-going screen, the number of wild-type controls can be minimised (Schofield et al., 2012). The current fiscal cost of pathology per mouse has been estimated at around US$300 (Adissu et al., 2014; Schofield et al., 2012), which includes the cost of processing the tissues to prepared slides and histopathology interpretation by a pathologist. This represents ~2.5% of the cost of production and *in vivo* phenotyping for a mouse line (based on pathology of four animals and a cost/line of approximately US$50,000), which provides good value for the amount of information potentially gained. This fact is not lost on the pharmaceutical industry, where pathology is still used routinely as a primary ‘phenotyping tool’ in research and development programmes (Schofield et al., 2012).

## Technical resources for pathology

Once included in a study plan, the next barrier to adoption of pathology is access to the appropriate technical resources. The reliance by the pharmaceutical industry on pathology means that there are extensive published standardised protocols for all steps in the pathology process, including systematic sampling of rodent tissues and data recording (Scudamore, 2014) [International Harmonization of Nomenclature and Diagnostic Criteria for Lesions (INHAND): https://www.toxpath.org/inhand.asp]. Dissection skills can be easily learnt and standard fixatives such as formalin, which is suitable for most routine morphological applications, are cheap (e.g. US$1/500 ml ready-made formalin) in comparison to the costs of generating and breeding a new mouse line. Recording of macroscopic findings to standardised glossaries is also a well-used tool, whether using traditional pathology descriptors or MPath ontological terms that refer to the size, shape, distribution, colour, consistency and quantity of lesions observed (Scudamore, 2014; Schofield et al., 2012). Once tissues have been harvested, the next step in good pathology is to prepare consistent sections. Although histological techniques are skills that can be learnt, most countries have contract histology laboratories who offer this as a paid service (costing around US$10–12/slide) to provide fast, reproducible and quality-controlled results. Good preparation, basic skills training and sensible use of outsourcing for specialist techniques should allow any group to collect samples, record gross observations and create slides suitable for accurate histopathological analysis.

## Accessing pathology expertise

Assuming pathology is included in the experimental plan from the start and the technical and cost hurdles surmounted, the ultimate challenge is to create meaningful data from the gross pathology observations and histopathology. Contrary to what has often been suggested (Warren et al., 2009), there might not be an actual lack of mouse pathology expertise globally, but rather a lack of available posts in academia for mouse pathologists. There are many toxicological pathologists (1200 in the US alone, 145 in US academia) (Turner et al., 2014), all of whom, already having experience of mouse histology, genetically modified mice and common background pathology, should be competent to analyse mouse tissues. The educational background of such pathologists should allow them to rapidly develop any additional expertise in mouse genetics, strain variation and an understanding of human disease relevance if they are not already familiar with these areas. The creation of positions for and attraction of pathologists to academic research should be a long-term goal for policy makers and budget holders if this experience gap is to be filled. In the meantime, the toxicological pathologists represent a considerable pool of expertise that can be tapped in to. Most trained laboratory animal pathologists are competent to make at least a primary assessment of all mouse tissues and it is arguably better for a single pathologist to evaluate all tissues from an animal in a primary screen, as is described in the paper by Adissu et al. (Adissu et al., 2014). This approach helps ensure that pleiotropic gene effects are detected and that full advantage is made of the pathologist’s experience in viewing tissues in a holistic way in combination with *in vivo* phenotyping data (Cardiff et al., 2008).

An experienced in-house pathologist who can contribute to experimental design as well as pathology interpretation should be considered best practice for centres running major phenotyping programmes. However, in smaller centres without direct access to a trained pathologist, accessing pathology expertise can be more challenging. The choices are: to contract out the pathology review to a collaborating pathologist or a private consultant laboratory animal pathologist, or obtain a basic level of training, preferably with on-going support, from an experienced pathologist. The use of telepathology can make expertise accessible even if it is at a distant centre. Laboratory animal pathologists work in academia, industry (CRO and Pharma) and as independent consultants, but there is no central register of contact details so some networking might be required to find expert assistance. Advice on how to contact pathologists can be gained via veterinary pathology departments in universities, toxicological pathology societies [STP (www.toxpath.org); ESTP (www.eurotoxpath.org); BSTP (www.BSTP.org.uk)] or industry contacts. Moreover, networks of comparative pathologists are starting to form on social networking sites such as LinkedIn (for example, the Comparative Pathologist Consortium). As with any other service, the cost of pathology expertise should be included in grant proposals to ensure that it is adequately budgeted for. Income generated in this way by the organisations employing pathologists is often used to provide the resource to train future pathologists and thereby sustain the expertise in this area.

In the event that a researcher needs to do their own pathology, they can be helped to gain expertise by attending courses run by the toxicologic pathology societies or academic institutes, or by taking specifically tailored courses online [e.g. Johns Hopkins Phenotyping and Pathology Core (http://www.hopkinsmedicine.org/mcp/PHENOCORE/); Centre for Genomic Pathology (http://ctrgenpath.net/); CL Davis foundation (www.cldavis.org)]. These courses allow not only basic training and knowledge transfer but also access to the pathologists providing the training and their (often global) network of pathology contacts. Skills that need to be learnt include the accurate recognition of normal tissue variation, the cause and appearance of technical artefacts, understanding of pathological lesions (spontaneous and induced), and the use of standardised glossaries and semi-quantitative scoring systems (Scudamore, 2014) to allow data generation. Common background and spontaneous pathologies are well described (and illustrated) for rodents, including mice [INHAND (https://www.toxpath.org/inhand.asp)] and for specific mouse strains (for example, Brayton et al., 2012). The nomenclatures produced by the toxicological pathologists represent the distillation of many years of pathology experience and so offer a robust route for recognising common lesions. These nomenclatures can be readily translated to ontological notation (Schofield et al., 2012) if required and ontological terms can be used to describe new phenotypic appearances. Ideally, pathology findings should be reviewed by an experienced pathologist at some time in the process and at least at the time of publication to avoid inaccurate data and conclusions from entering the literature. There is an onus on editors of scientific journals to ensure that pathology data that is presented in manuscripts is reviewed by people with appropriate expertise.

So, how can we eat the elephant of mouse pathology? As the saying goes, ‘one bite at a time’. Firstly we must start by doing what we can with the resources available and by looking outside the world of mouse phenotyping for the solutions that are already present. We need to make best use of those experts that are available in the phenotyping community and in the wider pathology community to disseminate knowledge, training and best practice. Journal editors and grant reviewers should ensure that pathology end points are not only included in manuscripts and applications but that these are supported by appropriate expertise and are adequately funded. Ultimately, funding organisations, universities and research institutes need to create career structures that allow expert mouse pathologists to work within academic organisations if this skills gap is to be closed.
